# Hyperpolarization of [1‐^13^C]Ketoisocaproate‐d_2_ by Reversible Exchange with Parahydrogen Enables Profiling of Branched‐Chain‐Amino‐Acid Metabolism in Cellulo and in Vivo

**DOI:** 10.1002/advs.76213

**Published:** 2026-06-23

**Authors:** Stefan Petersen, Philipp R. Groß, Paul M. Schmidt, Henri de Maissin, Asitan Rittinger, Robert Willing, Adriana Sacristán‐Martín, Lisa Heß, Jule Koch, Sebastian Lucas, Maxim Zaitsev, Dominik von Elverfeldt, Martin Grashei, Franz Schilling, Jan‐Bernd Hövener, André F. Martins, Max von Delius, Thomas Reinheckel, Andreas B. Schmidt

**Affiliations:** ^1^ Division of Medical Physics Department of Radiology Medical Center ‐ University of Freiburg Faculty of Medicine ‐ University of Freiburg Freiburg Germany; ^2^ German Cancer Consortium (DKTK) partner site Freiburg a Partnership Between DKFZ and University Medical Center Freiburg Heidelberg Germany; ^3^ Faculty of Biology Albert Ludwigs University Freiburg Freiburg Germany; ^4^ Institute of Organic Chemistry and Center for Integrated Quantum Science and Technology (IQST) University of Ulm Ulm Germany; ^5^ Institute of Molecular Medicine and Cell Research Faculty of Medicine University of Freiburg Freiburg Germany; ^6^ NVision Imaging Technologies GmbH Ulm Germany; ^7^ Technical University of Munich TUM School of Medicine and Health Department of Nuclear Medicine TUM University Hospital Munich Germany; ^8^ German Cancer Consortium (DKTK), partner site Munich a Partnership Between DKFZ and TUM Heidelberg Germany; ^9^ Section Biomedical Imaging Molecular Imaging North Competence Center MOINCC Department of Radiology and Neuroradiology University Hospital Schleswig Holstein Kiel University Kiel Germany; ^10^ Werner Siemens Imaging Center Department of Preclinical Imaging and Radiopharmacy Eberhard Karls University Tübingen Tübingen Germany; ^11^ Cluster of Excellence iFIT (EXC 2180) “Image‐Guided and Functionally Instructed Tumor Therapies” Eberhard Karls University Tübingen Tübingen Germany; ^12^ German Cancer Consortium (DKTK), partner site Tübingen a Partnership Between DKFZ and University Hospital Tübingen Heidelberg Germany; ^13^ Centre for Biological Signalling Studies BIOSS University of Freiburg Freiburg Germany

**Keywords:** hyperpolarization, ketoisocaproate, metabolic imaging, parahydrogen, SABRE

## Abstract

Hyperpolarized ^1^ ^3^C magnetic resonance imaging (MRI) is the only method to image metabolic fluxes in real time, non‐invasively, and in vivo. To date, however, most studies have used [1‐^1^ ^3^C]pyruvate and dynamic nuclear polarization (dDNP). Here, we establish efficient hyperpolarization (HP) of protio and partially‐deuterated [1‐^1^ ^3^C]ketoisocaproate (KIC) using Spin‐Lock‐Induced‐Crossing‐Signal Amplification by Reversible Exchange (SLIC‐SABRE), a high‐throughput, uncomplex and low‐cost method based on parahydrogen. We demonstrate ^13^C polarization up to ≈28% and T_1_ relaxation times > 200 s at 1 T in methanol‐d_4_. A rapid purification procedure allowed us to obtain biocompatible formulations with ≈11% ^13^C polarization at the time of injection, sufficient for in cellulo and in vivo studies. We found that branched‐chain‐amino‐acid transaminase (BCAT) activity leads to HP [1‐^1^ ^3^C]leucine formation exclusively in BCAT1‐high MDA‐MB231 breast‐cancer cells, but not in BCAT1‐low MCF7 or PyB6 cells. In a proof‐of‐concept in vivo experiment, ^13^C magnetic resonance spectroscopy imaging of the healthy mouse brain detected [1‐^13^C]leucine formation after intravenous injection of SABRE [1‐^13^C]KIC‐d_2_. Our results demonstrate that [1‐^13^C]KIC‐d_2_ provides a sensitive readout of BCAT1‐dependent metabolism and that SLIC‐SABRE can rapidly generate this probe for ^13^C MRI, extending accessible parahydrogen‐based hyperpolarization to amino‐acid pathways relevant to cancer biology and chemoresistance.

## Graphical abstract

We report the fast generation of biocompatible solutions of highly ^13^C‐hyperpolarized (≈11%) partially‐deuterated ketoisocaproate within <6 min using spin‐lock‐induced crossing and reversible exchange with parahydrogen (SLIC‐SABRE). This enables rapid detection of branched‐chain‐amino‐acid transferase 1 (BCAT1) protein expression and investigation of leucine formation in breast cancer cells and healthy mouse brain.

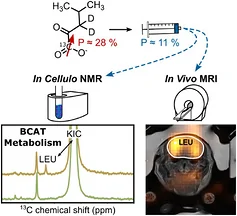

## 1 Introduction

Molecular imaging provides powerful insights into cancer biology by enabling non‐invasive assessment of metabolic reprogramming and biomarkers for treatment response [1, 2]. Positron emission tomography (PET) has transformed oncology, most prominently through^18^F‐fluorodeoxyglucose, and a broad portfolio of PET tracers is clinically established or under active development for imaging receptors, transporters, and metabolic processes [3]. However, PET exposes patients to ionizing radiation, which can limit repeated follow‐up examinations and application in some patient groups. Moreover, PET typically reports tracer uptake, distribution, or target binding, whereas direct real‐time monitoring of the chemical conversion of an injected metabolic substrate into downstream products is generally not accessible.

Hyperpolarized (HP) ^13^C magnetic resonance imaging (MRI) is a complementary approach and the only method allowing imaging of metabolic fluxes non‐invasively, in vivo, and in real time. HP MRI uses >10 000‐fold enhanced sensitivity with metabolic specificity to monitor metabolism in 3D with a temporal resolution of seconds without ionizing radiation [4]. The most clinically advanced tracer is [1‐^13^C]pyruvate, hyperpolarized by dynamic nuclear polarization (dDNP). More than 50 clinical trials have demonstrated its potential for diagnostics and therapy‐response assessment [5, 6, 7, 8]. The broader clinical adoption of HP MRI will ultimately depend on whether it provides actionable information that improves patient management. In parallel, the complexity, cost, and limited throughput of current dDNP technology remain relevant practical challenges, particularly for widespread preclinical implementation and multi‐center accessibility [9]. Parahydrogen (pH_2_) based hyperpolarization methods, including Signal Amplification by Reversible Exchange (SABRE), offer rapid and low‐cost alternatives to dDNP and have attracted considerable interest due to their low hardware demands, operational simplicity, and short polarization times [10]. In SABRE, pH_2_ and a substrate reversibly coordinate at an iridium N‐heterocyclic carbene complex so that the pH_2_ spin order can be transformed to polarization on the substrate. A major breakthrough was achieved in 2018, when pyruvate was first successfully hyperpolarized with SABRE‐SHEATH (SABRE in Shield Enable Alignment Transfer to Heteronuclei) [11, 12]. This proof‐of‐concept initiated substantial progress in SABRE theory, chemistry, and polarization‐transfer methodology [13, 14, 15, 16], and markedly higher pyruvate ^13^C polarization levels [17, 18]. A notable development was the application of Spin‐Lock Induced Crossing SABRE (SLIC‐SABRE) [19] at microtesla fields [20, 21, 22], and in particular its combination with substrate perdeuteration, which yielded significantly higher polarization levels for pyruvate in some studies [20, 23]. A major obstacle remained the generation of biocompatible, highly polarized injectable formulations, as SABRE typically involves organometallic catalysts and organic solvents. Considerable chemical effort has therefore focused on purification strategies including catalyst scavenging, phase separation, and solvent evaporation, although additional work is needed to meet routine preclinical and clinical requirements [23, 24, 25, 26, 27, 28]. This cumulative progress ultimately enabled the first in vivo metabolic ^13^C MRI studies with SABRE‐hyperpolarized pyruvate in mice, including wildtype [26, 27, 29] and cancer models [30, 31, 32].

Hyperpolarized [1‐^13^C]pyruvate is the most widely used probe to image metabolism in real time in vivo, as many pathologies, including cancer, exhibit alterations in pyruvate metabolism [33]. However, because rapidly proliferating glycolytic tumors often respond well to chemotherapy [34], additional probes with higher pathway specificity are needed to better capture the metabolic diversity of cancer, supporting therapy selection [35, 36, 37, 38].

One promising candidate is ketoisocaproate (KIC), which can be converted to leucine (LEU) by branched‐chain amino acid aminotransferases (BCATs) [39]. BCAT activity is upregulated in several cancers and is linked to tumor proliferation and metastasis [40, 41]. Importantly, altered branched‐chain amino acid metabolism has also been associated with chemoresistance in acute myeloid leukemia [42]. Prior dDNP studies have demonstrated the feasibility of detecting HP KIC metabolism in vitro [43] and in vivo [39, 44, 45, 46]. Other studies have demonstrated that KIC can be hyperpolarized using SABRE [47, 48] and that biocompatible solutions can be obtained for monitoring metabolism in yeast [49]. Yet the achievable ^13^C polarization after purification remained at ≈3%, and higher polarizations are favorable for preclinical in vivo imaging. To date, no in vivo experiments with SABRE‐polarized KIC have been reported.

Here, we address several of these key physicochemical challenges associated with in vivo MRI with SABRE hyperpolarized KIC. By combining a tailored SLIC transfer scheme with selected deuterium labeling, we achieved up to 28% polarization for [1‐^13^C]KIC‐d_2_ prior to purification. We present a rapid purification that yields biocompatible KIC formulations with ≈11% ^13^C polarization, and demonstrate that it can resolve differences in BCAT activity across breast cancer cell lines with distinct expression levels. Finally, we present the first in vivo conversion of SABRE‐polarized KIC to LEU using ^13^C MRSI in the healthy mouse brain.

## 2 Results and Discussion

### 2.1 SABRE Hyperpolarization of KIC

Protio‐ or partially‐deuterated KIC (custom‐synthesized, Figures S1–S7), iridium–NHC precatalyst IrCl(COD)(IMes), DMSO‐d_6_ and EDTA were dissolved in methanol‐d_4_, transferred to a 5 mm NMR tube and centered in a home‐built SABRE polarizer comprising µ‐metal shielding, static and radiofrequency field coils, and water‐based cooling (Figure S8). Continuously supplying pressurized pH_2_ gas (90% enrichment) activated the catalyst and enabled reversible coordination and polarization transfer at SLIC‐SABRE or SABRE‐SHEATH magnetic field conditions (Figure 1a; Figure S8b).

**FIGURE 1:**
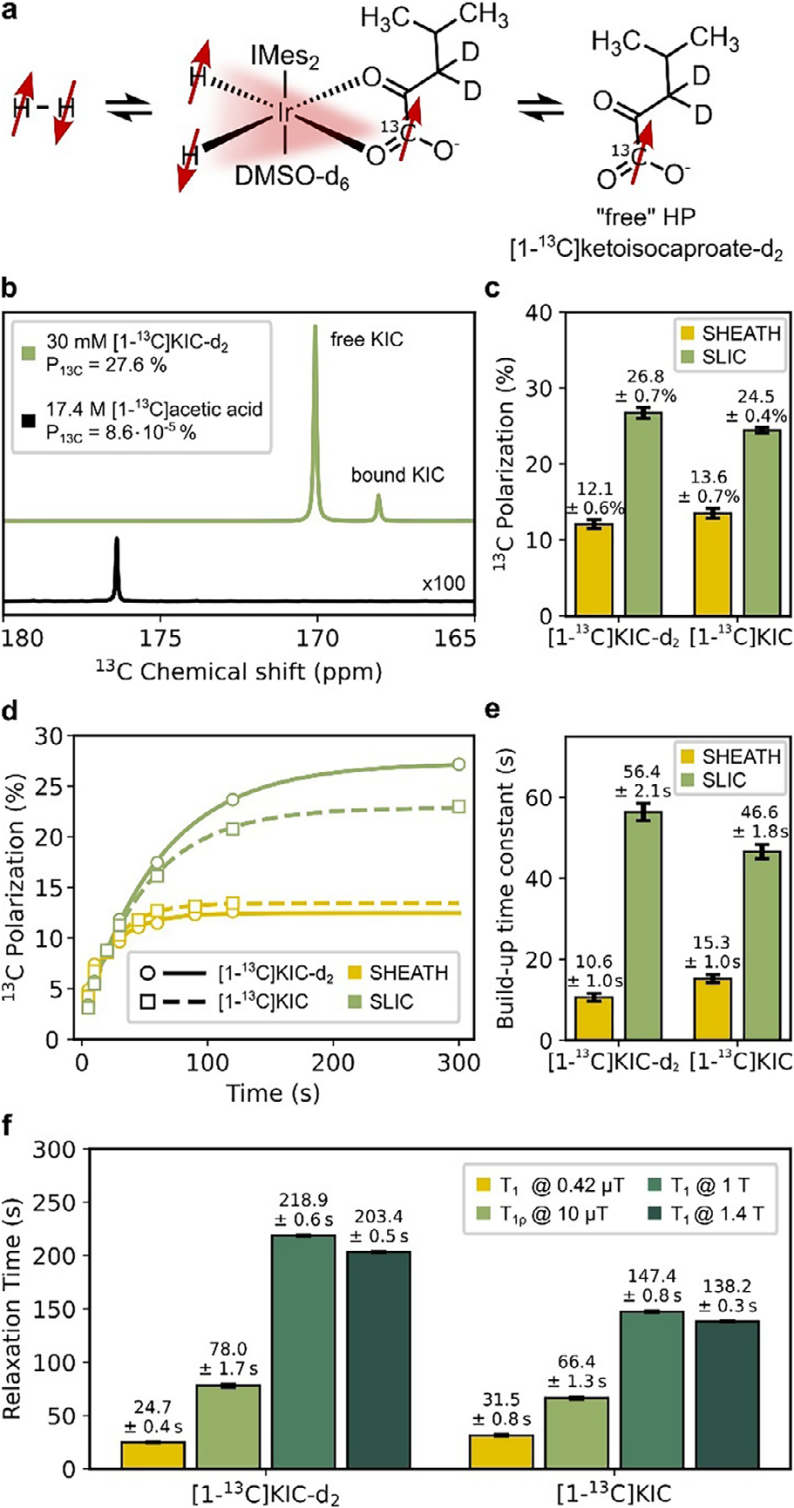
Efficient SLIC‐SABRE hyperpolarization of [1‐^13^C]ketoisocaproate ([1‐^13^C]KIC). (a) Schematic SABRE reaction showing reversible coordination of pH_2_ and [1‐^13^C]KIC‐d_2_ to the Ir complex. (b) ^13^C NMR showing 27.6% ^13^C polarization of 30 mm [1‐^13^C]KIC‐d_2_ in comparison to a 17.4 M reference solution of neat [1‐^13^C]acetic acid at 1 T. (c) Average of highest ^13^C polarization levels achieved for the two isotopologues using SABRE‐SHEATH (0.42 µT) or SLIC‐SABRE (at 10 µT). (d) ^1^ ^3^C polarization after varied build‐up times and fit used to extract (e) build‐up time constants for protonated and partially deuterated [1‐^1^ ^3^C]KIC under SABRE‐SHEATH and SLIC‐SABRE conditions. (f) Field‐dependent ^1^ ^3^C T_1_ constants in methanol‐d_4_ at 0.42 µT, 1.0 T and 1.4 T, and ^13^C T_1ρ_ constant during SLIC (i.e., in the presence of an oscillating B_1_ field at 10 µT). Except for sub‐µT fields, deuteration reduced relaxation. Error bars represent the standard deviation from *N* = 3 independent measurements.

We obtained high and reproducible ^13^C polarization of up to ≈27.6 % for 30 mm [1‐^13^C]KIC‐d_2_ by using a dedicated SLIC‐SABRE protocol at 10 µT (26.8 ± 0.7 % on average, *N* = 3; Figure 1b). These polarization levels were achieved after a systematic exploration of key transfer parameters, including static field strength (Figure S9), SLIC pulse conditions (Figure S10), reaction temperature (Figure S11), and deuterium labeling (Figure S1).

For comparison, we conducted SABRE‐SHEATH experiments and found that SLIC‐SABRE yielded approximately two‐fold higher ^13^C polarization for both isotopologues (Figure 1c). We attribute the boost in polarization to the longer time available for spin‐order build‐up under microtesla SLIC conditions (Figure 1d,e). By varying the polarization transfer time, we found that for [1‐^13^C]KIC‐d_2_, the build‐up time constant increased from 10.6 ± 1.0 s for SHEATH to 56.4 ± 2.1 s for SLIC.

These findings correlated well with measured relaxation constants: the relaxation time constant under SLIC conditions at 10 µT reached T_1ρ_ = 78.0 ± 1.7 s for [1‐^13^C]KIC‐d_2_, compared to T_1_ = 31.5 ± 0.8 s for protonated [1‐^13^C]KIC and T_1_ = 24.7 ± 0.4 s for [1‐^13^C]KIC‐d_2_ under SABRE‐SHEATH conditions at 0.42 µT (Figure 1f). Partial deuteration additionally extended high‐field polarization lifetimes (e.g., T_1_ at 1 T of 218.9 ± 0.6 s for [1‐^13^C]KIC‐d_2_ versus 147.4 ± 0.8 s for protonated [1‐^13^C]KIC) in methanol‐d_4_, favorable for purification and handling of HP KIC (Figure 1f; Figure S12). This effect can be attributed to reduced proton‐driven dipole–dipole relaxation, a major relaxation pathway for ^13^C nuclei in solution [50].

These features of KIC are consistent with observations for pyruvate, where SLIC‐SABRE and deuteration were found to improve ^13^C polarization, too. However, the polarization of KIC even exceeds that reported for HP pyruvate under comparable conditions [20]. In this earlier work, notably on deuterated pyruvate, it has been observed that deuterons can act as quadrupolar relaxation sinks at sub‐µT fields, leading to strongly shortened T_1_ and markedly reduced SABRE‐SHEATH polarization [20, 51]. At such ultralow fields, deuterons can become coupled to the same spin network that mediates heteronuclear polarization transfer, so that part of the pH_2_‐derived spin order is dissipated through fast quadrupolar relaxation instead of being retained as ^13^C polarization. This detrimental pathway is much less relevant at higher magnetic fields, where the beneficial reduction of proton‐driven dipole–dipole relaxation dominates. Here, this detrimental effect, however, was less pronounced for KIC. This difference may arise from the different labeling pattern – KIC‐d_2_ is only partially deuterated at the C3 position – or from intrinsic structural differences, such as the methyl group in pyruvate compared with the methylene/methyl pattern in KIC. Thus, while deuteration remains beneficial for SLIC‐SABRE of both pyruvate and KIC, its impact on polarization efficiency appears to be substrate‐dependent and warrants further investigation.

### 2.2 Producing Biocompatible Formulations

Motivated by the high polarization levels obtained above, we next established a rapid purification protocol that removed ≈99% of methanol and iridium catalyst within 30 s, yielding KIC‐d_2_ solutions biocompatible for in vitro and in vivo applications (Figure 2a). In detail, the methanol solvent was replaced by phosphate‐buffered saline (PBS) in D_2_O through a novel two‐step procedure lasting 16 s (Figure S13): First, 300 µL of buffer was added under vacuum (10 mbar) while the sample was placed in a boiling water bath and an elevated magnetic field (100 µT). Eleven seconds after the start of injection, an additional 300 µL of buffer was added, ensuring reproducibly low methanol concentrations. Subsequently, the iridium catalyst, which precipitated due to its low water solubility, was removed by passing the solution through a microporous syringe filter prior to in vitro and in vivo experiments.

**FIGURE 2:**
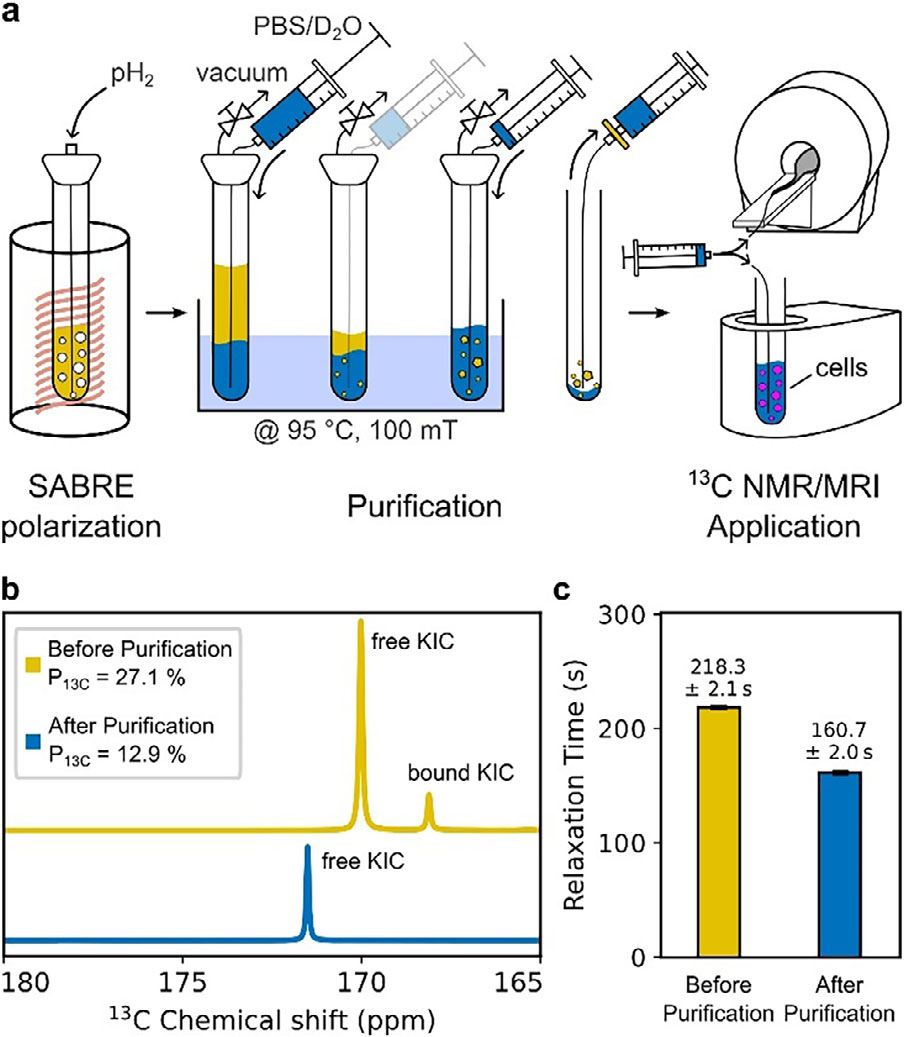
(a) Schematic showing the procedure used to obtain biocompatible 50 mm [1‐^13^C]KIC‐d_2_ solutions. (b) ^13^C NMR spectra showing the ^13^C polarization before and retention after purification. The purified samples (283 ± 27 µL) were diluted with 200 µL cell medium. (c) ^13^C T_1_ relaxation times at 1 T for a [1‐^13^C]KIC‐d_2_ solution before purification in methanol‐d_4_ and after purification in PBS in D_2_O. Error bars represent the standard error obtained from the fit.

For cell experiments, a hyperpolarized 10 mm KIC‐d_2_ solution was processed, and we obtained an aqueous solution of 283 ± 27 µL with pH 7.4, a final concentration of c_KIC_ = 16.3 ± 1.0 mm and a polarization of P(^13^C) = 9.5 ± 0.5% (preserving about 0.44x the initial polarization, Figure S14a). For in vivo experiments, where higher concentrations are required, a hyperpolarized aqueous solution of ≈50 mm KIC‐d_2_ with a polarization of P(^13^C) = 10.4 ± 0.6% was produced, exhibiting otherwise comparable properties (290 ± 17 µL, pH 7.4, Figure 2b). The final solutions were analyzed via ^13^C NMR, ^2^H NMR and ICP‐OES (Figures S14 and S15), and the results are summarized in Table S1.

Notably, the remaining methanol‐d_4_ content did not exhibit apparent cytotoxicity [52], particularly after dilution in cell medium or the bloodstream in vivo (Figure S18), supporting the suitability of the formulation for pioneering preclinical applications. Importantly, the purified sample maintained a long T_1_ at 1 T of 160.7 ± 2.0 s, facilitating sample handling and administration between purification and ^13^C NMR/MRI application (Figure 2c; Figure S16).

### 2.3 Metabolic Profiling of Breast Cancer Cells

Given the strong polarization, the successful purification and the central role of KIC in branched‐chain amino acid metabolism (Figure 3a), we next examined whether SABRE‐HP KIC can sensitively report on differences in cytosolic BCAT activity. To this end, we selected three breast‐cancer cell lines with distinct BCAT1 and BCAT2 protein expression levels (Figure 3b; Figure S19): MDA‐MB231 cells (BCAT1‐high, BCAT2‐low), MCF7 cells (BCAT1‐low, BCAT2‐high), and PyB6 cells (BCAT1‐low, BCAT2‐low) [53].

**FIGURE 3:**
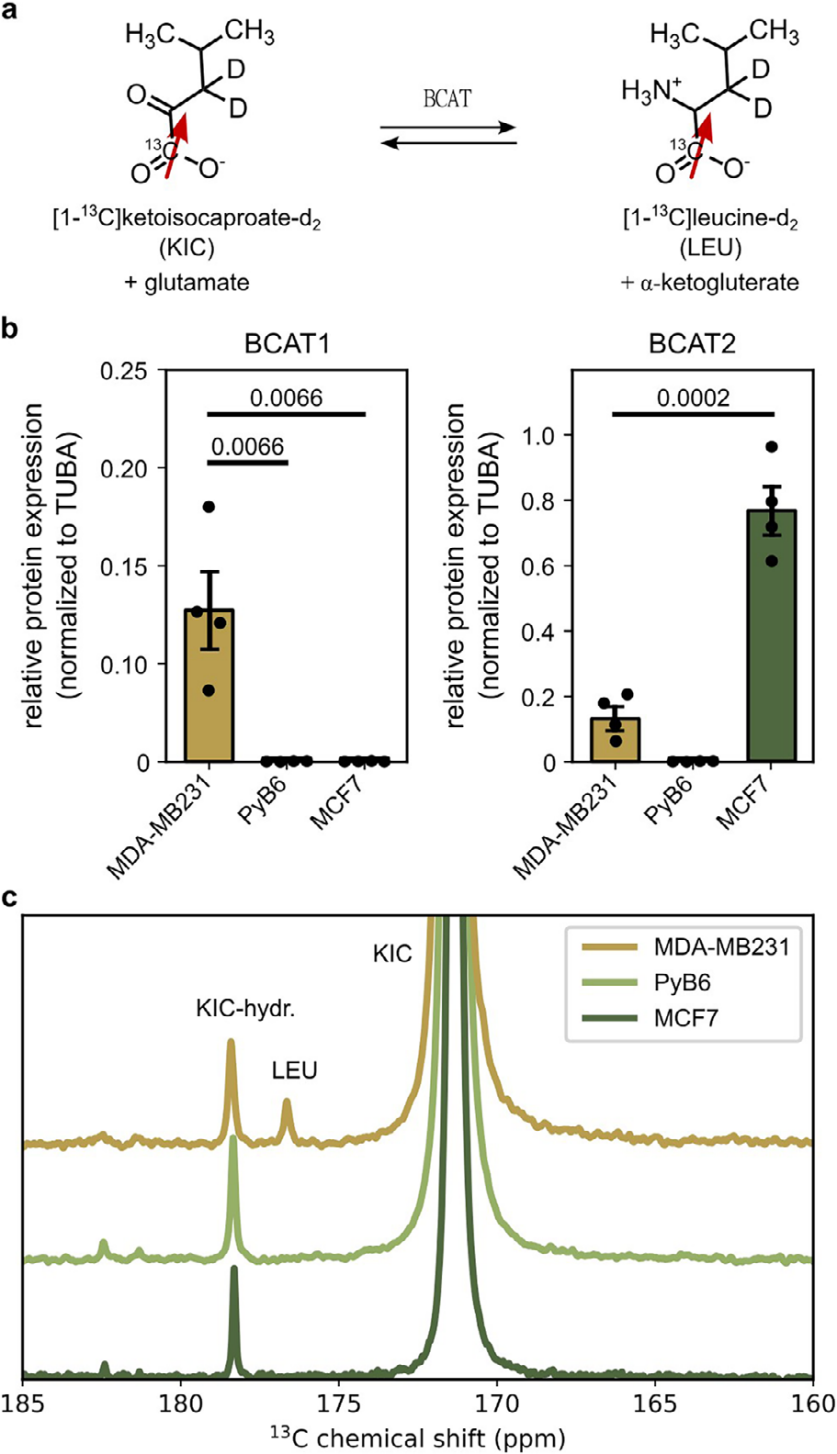
(a) Branched‐chain‐amino‐acid transaminase (BCAT) catalyzes the reversible conversion of ketoisocaproate (KIC) to leucine (LEU) and alpha‐ketoglutarate using glutamate as the amino donor. (b) BCAT1 and BCAT2 protein expression in MDA‐MB231, PyB6, and MCF7 cells (N = 4, unpaired t‐test). (c) ^13^C NMR spectra of hyperpolarized [1‐^13^C]KIC‐d_2_ injected into suspensions of breast cancer cells of different lines with high (MDA‐MB231) and low BCAT1 expression (PyB6; MCF7). 90 s post‐injection, an elevated LEU formation is detected in MDA‐MB231 cells. Weak resonances at ∼182 ppm and KIC‐hydrate at 178.3 ppm are present prior to cell addition and are not attributed to cellular metabolism.

Biocompatible [1‐^13^C]KIC‐d_2_ solutions were injected into 200 µL suspensions of 7.5 × 10^6^ cells and analyzed by a single 90° ^13^C NMR acquisition 90 s post‐injection at 1 T.

All cells showed the expected KIC resonance at 171.3 ppm. Most interestingly, however, only the MDA‐MB231 cells exhibited a new resonance at 176.7 ppm, corresponding to [1‐^13^C]LEU formation (Figure 3c). Two weak resonances around 182 ppm, as well as the resonance of the hydrated form of KIC at 178.3 ppm [49], were already present in purified hyperpolarized KIC solutions prior to addition to cells (Figure S17), indicating that they do not originate from cellular metabolism. We therefore assign them conservatively to minor KIC‐derived species formed during purification. Importantly, these resonances are spectrally separated from the [1‐^1^ ^3^C]leucine signal and do not affect the interpretation of BCAT‐mediated transamination.

LEU formation was detected exclusively in BCAT1‐high but not in BCAT2‐high cell lines. Therefore, the observed metabolic conversion predominantly reflects BCAT1 activity, which makes sense as the HP substrate accesses the cytosol far more rapidly than the mitochondrial matrix. Notably, probing BCAT1 non‐invasively may have significant relevance for predicting therapy resistance [42, 53].

### 2.4 In Vivo Experiments

These highly promising results lead us to examine whether SABRE‐HP KIC can also report on KIC‐to‐LEU metabolism in vivo. For this proof‐of‐concept experiment, solutions containing ≈50 mm hyperpolarized [1‐^13^C]KIC‐d_2_ were administered via tail‐vein catheter into a healthy C57Bl/6N mouse (10 µL/g mouse weight over ≈5 s), and ^13^C MR spectroscopy imaging (MRSI) was acquired from the mouse head (Figure 4a). While KIC was detected in the whole head with major signal contribution from the blood vessels, LEU signal was mainly detected from the brain.

**FIGURE 4:**
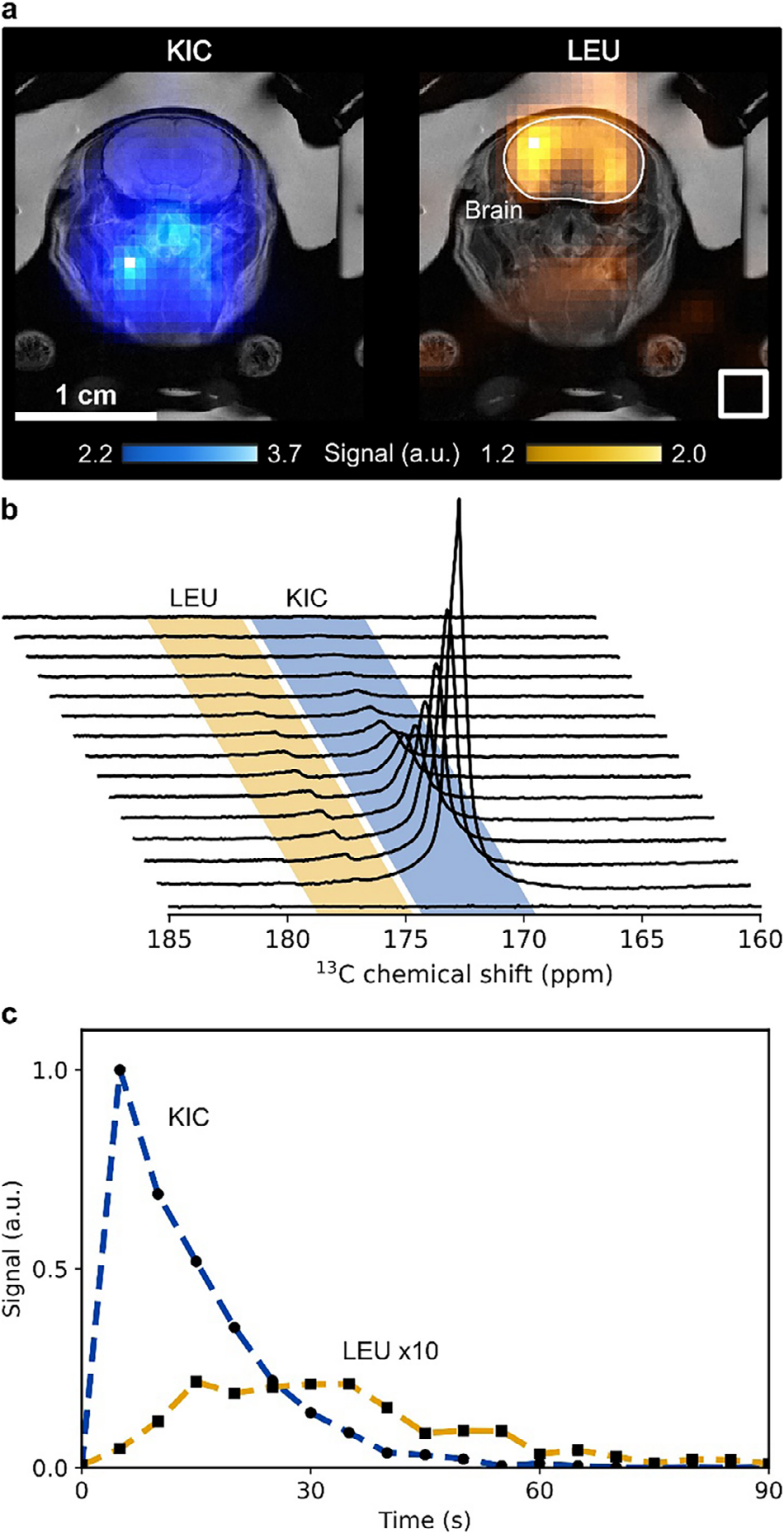
In vivo ^13^C MRSI of healthy mice after intravenous injection of 50 mm hyperpolarized KIC. (a) ^13^C chemical shift imaging was acquired starting 15 s post‐injection. 2D ^13^C metabolite maps of KIC and LEU are superimposed with an anatomical ^1^H MRI of the same axial slice, revealing LEU formation particularly in the brain. The white box (bottom right) indicates the native CSI‐FID resolution. ^13^C images without interpolation are provided in the SI (Figure S21). (b) ^13^C spectra acquired dynamically every 5 s using 15° flip angles show the injection of KIC, production of LEU, and decay of both signals. **c** Time courses of the metabolite signal amplitudes obtained by integration over the regions indicated in (b).

In a second experiment, following bolus injection with the mouse head centered in the RF coil, dynamically acquired global ^13^C NMR spectra revealed the emergence of a distinct [1‐^13^C]LEU resonance (Figure 4b). Notably, both peaks remained distinguishable despite the limited B_0_ field homogeneity in this example. The corresponding time courses obtained by spectral integration of the KIC and LEU peaks show clear in vivo formation of LEU, reaching a maximum approximately 20–35 s post‐injection (Figure 4c). The apparent T_1_ of [1‐^13^C]KIC‐d_2_ in vivo at 7 T was found to be 19.2 ± 1.6 s (Figure S20).

These data provide the first evidence that SABRE‐polarized KIC can reveal BCAT‐mediated metabolic conversion in vivo and demonstrate the robustness of this approach. The current formulation is suitable for preclinical proof‐of‐concept studies but does not yet meet the requirements for clinical application. Residual CD_3_OD was 370 ± 56 mm, corresponding to approximately 10700 ppm by mass in D_2_O, which is above the ICH Q3C limit of 3000 ppm for methanol as a Class 2 residual solvent. For a hypothetical 30 mL human bolus, the current CD_3_OD concentration would correspond to approximately 400 mg of residual methanol, exceeding the permitted daily exposure of 30 mg/day. Residual iridium would likewise require substantial reduction to meet the parenteral permitted daily exposure of 10 µg/day. At the measured residual concentration of 88 ± 11 µm Ir, a 30 mL bolus would contain approximately 500 µg Ir [54, 55]. Thus, while the present purification already enables first preclinical applications, future clinical translation will require further reduction or replacement of residual solvent, more efficient catalyst removal, sterile and pyrogen‐free formulation, validated batch‐release analytics, toxicological evaluation, and GMP‐compatible production [56].

In addition, the current purification workflow is partly manual and should be transferred into an automated fluidic process in future work. Automation would improve reproducibility and scalability, while also allowing systematic optimization of solvent and catalyst removal, timing, and formulation parameters under defined process conditions.

Together, our polarization levels achieved after purification (≈11%) and the long in vitro T_1_ at conventional MRI fields (>160 s at 1 T) indicate that SABRE is an efficient method to hyperpolarize KIC, which has strong potential for biomedical studies. Building on our demonstration of BCAT1 specificity and in vivo feasibility, further advances in purification, formulation, and field‐optimized acquisition strategies should unlock the full metabolic information accessible with SABRE‐polarized KIC. Ultimately, this method provides an accessible ^13^C MRI of dysregulated BCAT1 metabolism in cancer and other diseases.

## 3 Conclusion

Investigation of polarization transfer and rapid purification yielded biocompatible KIC‐d_2_ formulations with ≈11% ^13^C polarization within <6 min, enabling metabolic readouts in both breast cancer cell lines and in vivo mice.

Using these preparations, we demonstrated BCAT1‐dependent conversion of KIC to LEU in cancer cells and the first in vivo observation of SABRE‐polarized KIC to LEU metabolism in the living mouse brain.

These results show that KIC is a sensitive probe for altered branched‐chain amino acid metabolism and expand the portfolio of rapid SABRE hyperpolarization toward biologically and clinically relevant metabolic pathways. With continued advances in SABRE methodology and formulation chemistry, SABRE‐polarized KIC promises to offer a highly accessible tool for metabolic cell assays, preclinical research, and future translational efforts. Please find additional experimental details, and sample characterization, NMR, and relaxation data in the Supporting Information file. The authors have cited additional references within the Supporting Information [57, 58, 59, 60, 61, 62, 63].

## Author Contributions

**Henri de Maissin**: conceptualization, writing – review and editing, data curation. **Philipp R. Groß**: conceptualization, investigation, writing – review and editing. **Sebastian Lucas**: conceptualization, writing – review and editing, formal analysis. **Adriana Sacristán‐martín**: conceptualization, writing – review and editing, formal analysis, data curation. **Maxim Zaitsev**: conceptualization, writing – review and editing. **Paul M. Schmidt**: conceptualization, investigation. **Stefan Petersen**: conceptualization, writing – original draft, visualization, investigation, formal analysis, data curation. **Robert Willing**: conceptualization, writing – review and editing. **Martin Grashei**: conceptualization, writing – review and editing, data curation. **Asitan Rittinger**: conceptualization, investigation. **Lisa Heß**: conceptualization, writing – review and editing. **Dominik v. Elverfeldt**: conceptualization, writing – review and editing. **Franz Schilling**: conceptualization, writing – review and editing. **André F. Martins**: conceptualization, writing – review and editing. **Max von Delius**: conceptualization, writing – review and editing. **Jan‐Bernd Hövener**: conceptualization, writing – review and editing. **Andreas B. Schmidt**: conceptualization, funding acquisition, writing – original draft, supervision, project administration. **Jule Koch**: conceptualization, formal analysis. **Thomas Reinheckel**: conceptualization, writing – review and editing.

## Conflicts of Interest

The authors declare no conflicts of interest.

## Supporting information




**Supporting File**: advs76213‐sup‐0001‐SuppMat.pdf.

## Data Availability

The data that support the findings of this study are available from the corresponding author upon reasonable request.
