# Supplementary material for: Hyperpolarization of [1‐13C]Ketoisocaproate‐d2 by Reversible Exchange with Parahydrogen Enables Profiling of Branched‐Chain‐Amino‐Acid Metabolism in Cellulo and in Vivo
Source: Adv Sci (Weinh). 2026 Jun 23:e76213. Online ahead of print. doi: 10.1002/advs.76213 (PMC13336365; doi:10.1002/advs.76213)
Supplement: Supplementary file 1 — Supporting File: advs76213‐sup‐0001‐SuppMat.pdf. [file ADVS-9999-e76213-s001.pdf]

Supporting Information

Hyperpolarization of [1-^{13}C]Ketoisocaproate-d_2 by Reversible Exchange with Parahydrogen Enables Profiling of Branched-Chain-Amino-Acid Metabolism *In Cellulo* and *In Vivo*

Stefan Petersen, Philipp R. Groß, Paul M. Schmidt, Henri de Maissin, Asitan Rittinger, Robert Willing, Adriana Sacristán-Martín, Lisa Heß, Jule Koch, Sebastian Lucas, Maxim Zaitsev, Dominik v. Elverfeldt, Martin Grashei, Franz Schilling, Jan-Bernd Hövener, André F. Martins, Max von Delius, Thomas Reinheckel, Andreas B. Schmidt*

Corresponding author: andreas.schmidt@uniklinik-freiburg.de

Table of Contents

Experimental Procedures 2
- Synthesis 2
- Sample Preparation 6
- Experimental Setup 6
- Hyperpolarization and Purification Experiments 7
- Hyperpolarization Quantification 7
- Cell handling 7

^{13}C NMR measurements 9
- Hyperpolarization optimization 9
- Purification 10

In Vitro Experiments 13
- Cell lines 13
- Western Blot 13

In Vivo Experiments 14
- MRI Sequences and Data Processing 15

References 16

Experimental Procedures

Synthesis

The IrIMes(COD)Cl pre-catalyst was synthesized using an established procedure.[1]

The partial deuteration was achieved by dissolving sodium [1-^{13}C]isocaproate (0.5 g, 3.3 mmol) in 6.0 mL of D_2O. NEt_3 (1. mL, 7.2 mmol, 2.2 equiv.) was added and the mixture was heated at 65 °C. After 16 h, the solvent was evaporated under vacuum, and a white powder was isolated as the pure compound (0.49 g, 97%) **^{1}H NMR** (600 MHz, D_2O) δ = 2.02 (hept, J = 6.7 Hz, 1H), 0.87 (d, J = 6.7 Hz, 6H). **^{2}H NMR** (92 MHz, H_2O) δ 2.56 (d, J = 1.1 Hz, 1H). **^{13}C NMR** (151 MHz, D_2O) δ = 208.1 (d, J (^{13}C–^{13}C) = 61.6 Hz), 171.4, 47.5 (dtd, J (^{13}C–^{2}H) = 39.0, 19.9, 12.4 Hz), 24.1, 21.7. **HRMS (ESI-neg)**: $m{\cdot}z^{-1}$ = calc. for $^{13}C_1{}^{12}C_5H_7D_2O_3^-$: 132.0711, found: 132.0726.

The partial deuteration of natural abundance KIC was performed using the same procedure (yield: 97%). **^{1}H NMR** (600 MHz, D_2O) δ 2.11 (hept, J = 7.1 Hz, 1H), 0.96 (d, J = 6.7 Hz, 6H). **^{13}C NMR** (151 MHz, D_2O) δ 208.13, 171.40, 47.51 (p, J = 19.4 Hz), 24.12, 21.72. **HRMS (ESI-neg)**: $m{\cdot}z^{-1}$ = calc. for $C_6H_7D_2O_3^-$: 131.0677, found: 131.0692.

All commercially available chemicals were purchased from Sigma Aldrich and used without further purification.

NMR

NMR spectra of [1-^{13}C]ketoisocaproate-d_2 ([1-^{13}C]KIC-d_2) were recorded on a BrukerAvance 600 NEO spectrometer (^{1}H: 600 MHz, ^{2}H: 91 MHz, ^{13}C: 151 MHz) at room temperature (298 K). Chemical shifts (δ) are reported in ppm using residual solvent protons (^{1}H NMR: δ_H = 4.79 ppm for D_2O) as internal standard. The splitting patterns are designated as follows: d (doublet), hept (septet), and dtd (doublet of triplets of doublets). Coupling constants (J) correspond to proton–proton, carbon–carbon, or carbon–deuterium couplings.

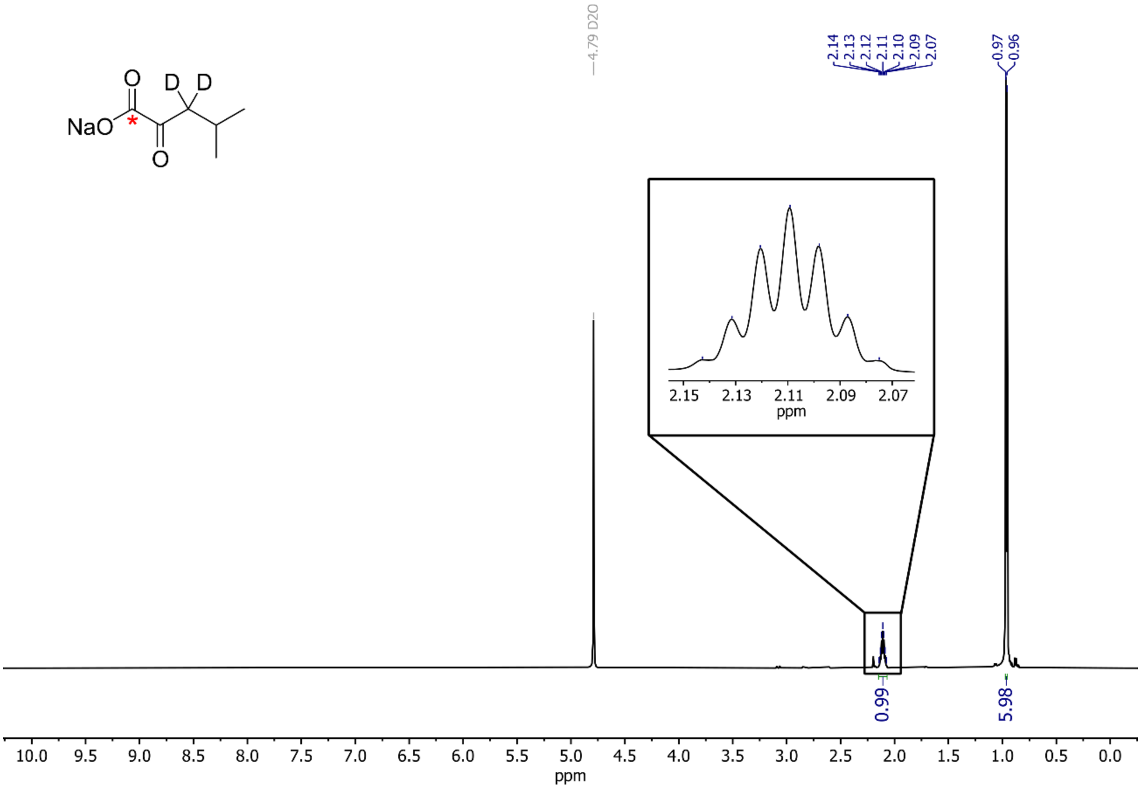

Figure S1: ^{1}H NMR (600 MHz, D_2O) of [1-^{13}C]KIC-d_2.

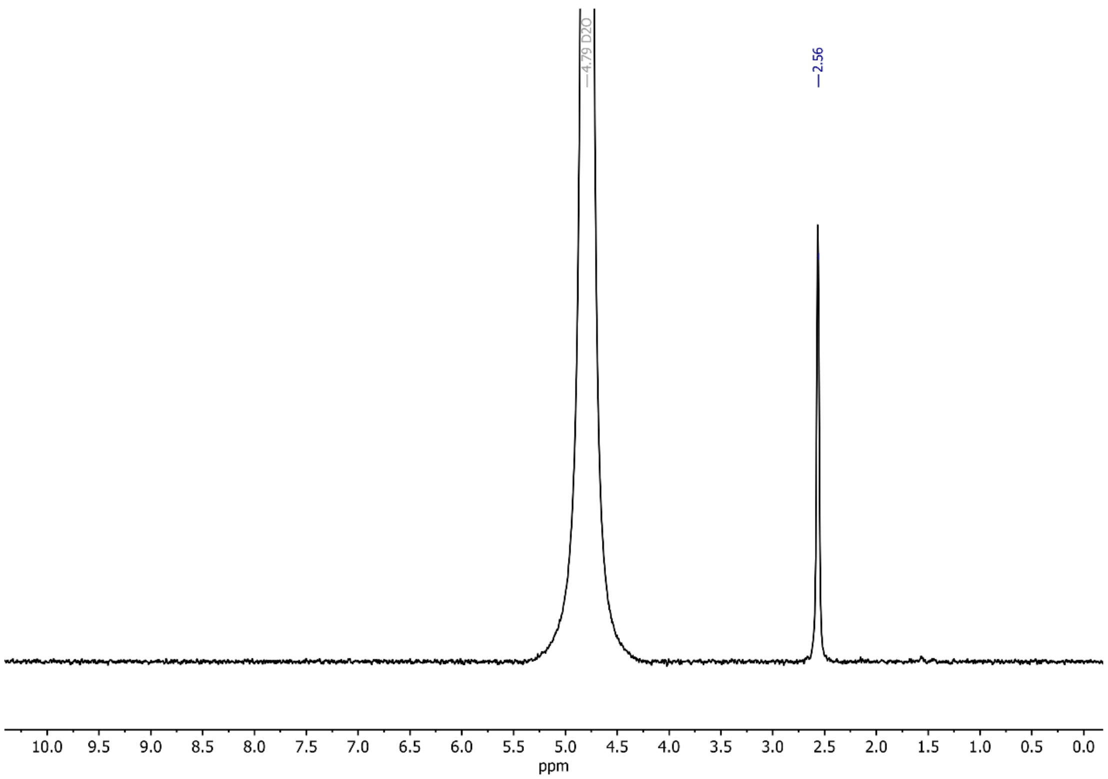

Figure S2: ^{2}H NMR (92 MHz, H_2O) of [1-^{13}C]KIC-d_2

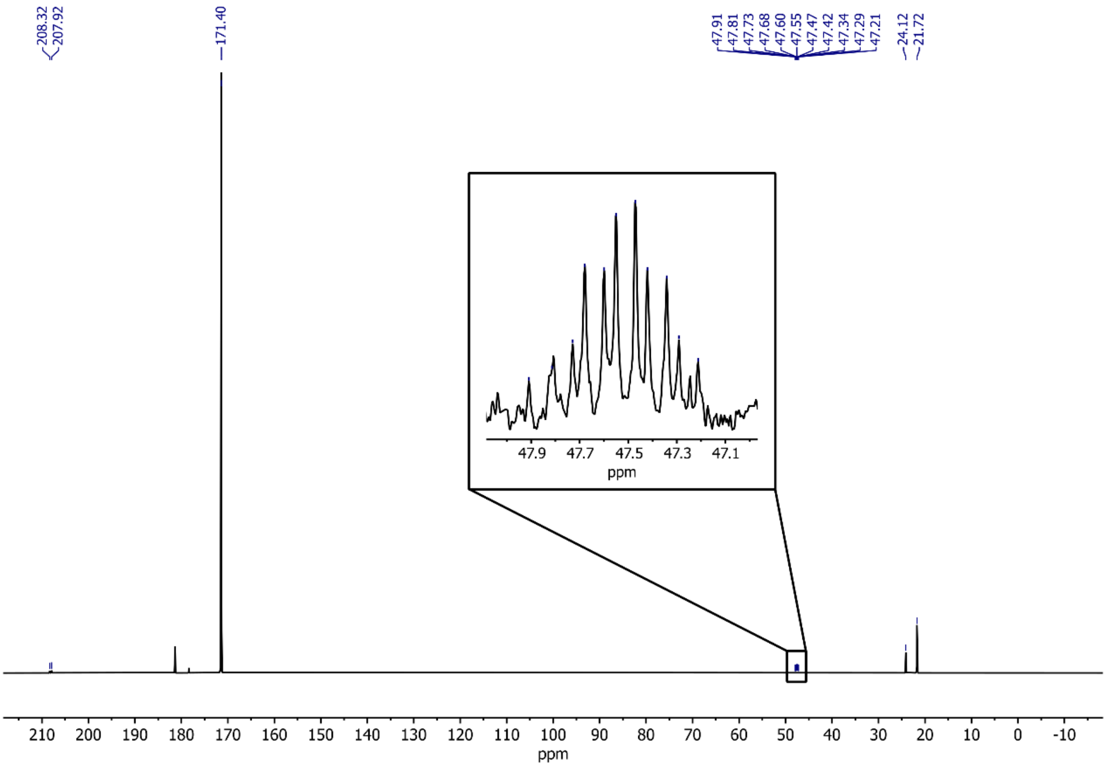

Figure S3: ^{13}C NMR (151 MHz, D_2O) of [1-^{13}C]KIC-d_2.

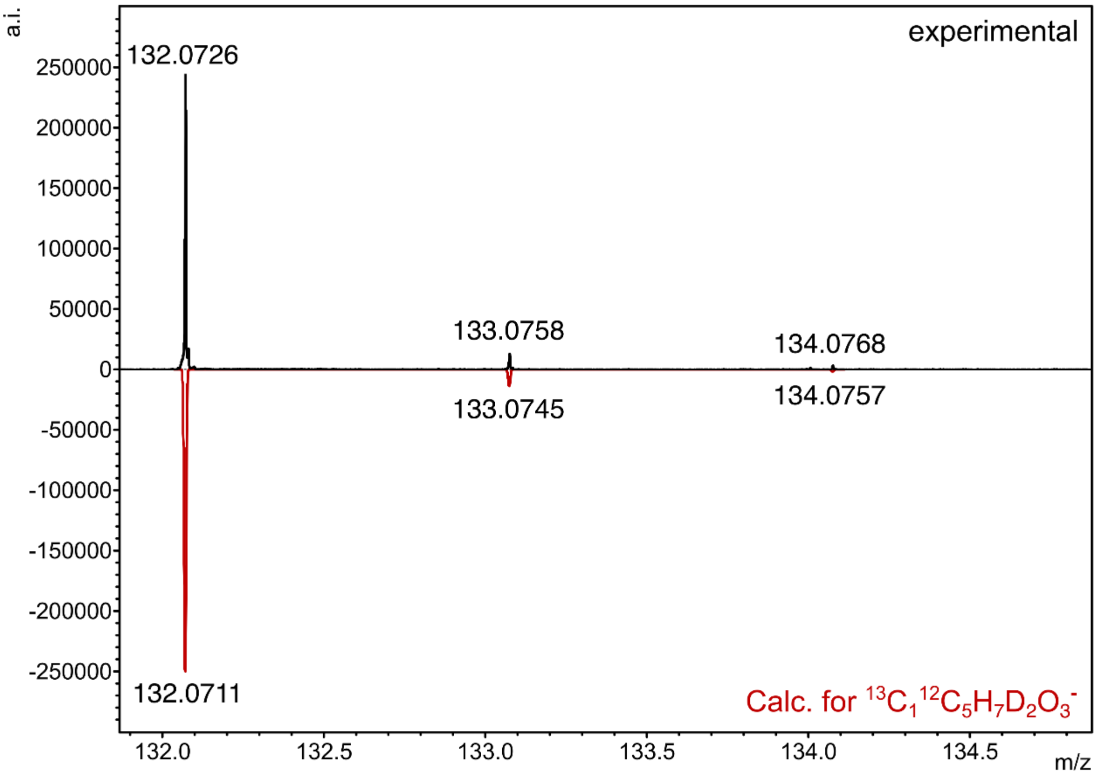

Figure S4: HRMS (ESI) of [1-^{13}C]KIC-d_2.

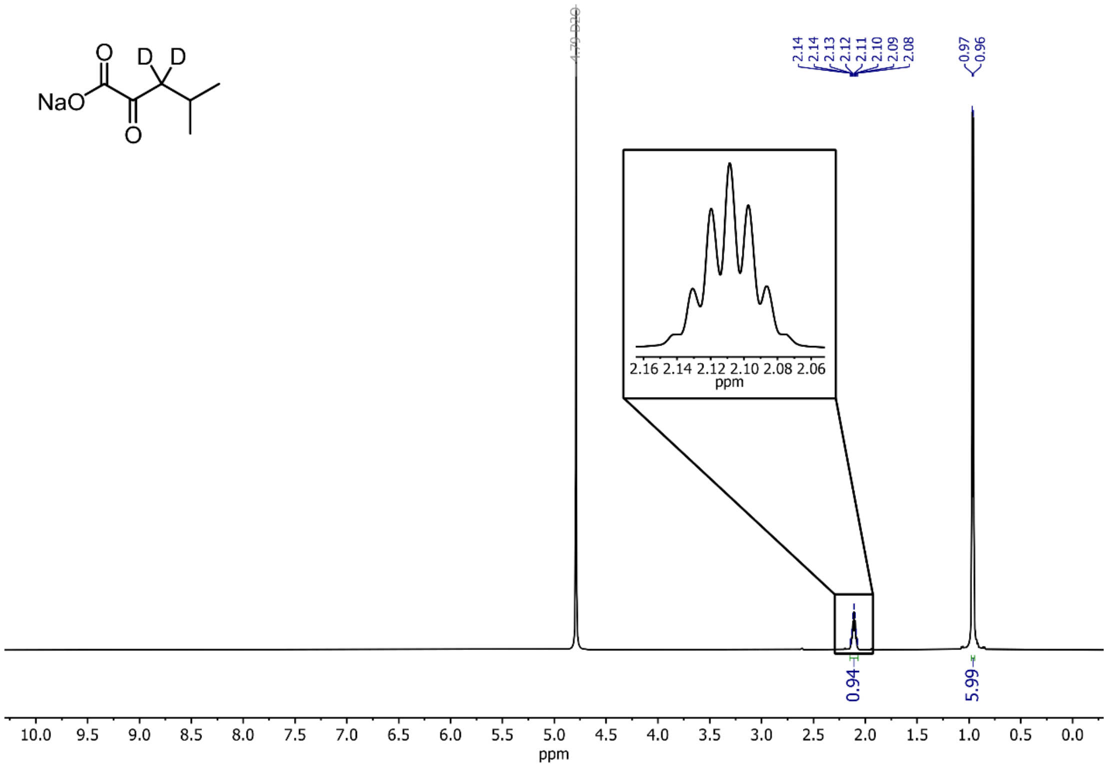

Figure S5: ^{1}H NMR (600 MHz, H_2O) of KIC-d_2.

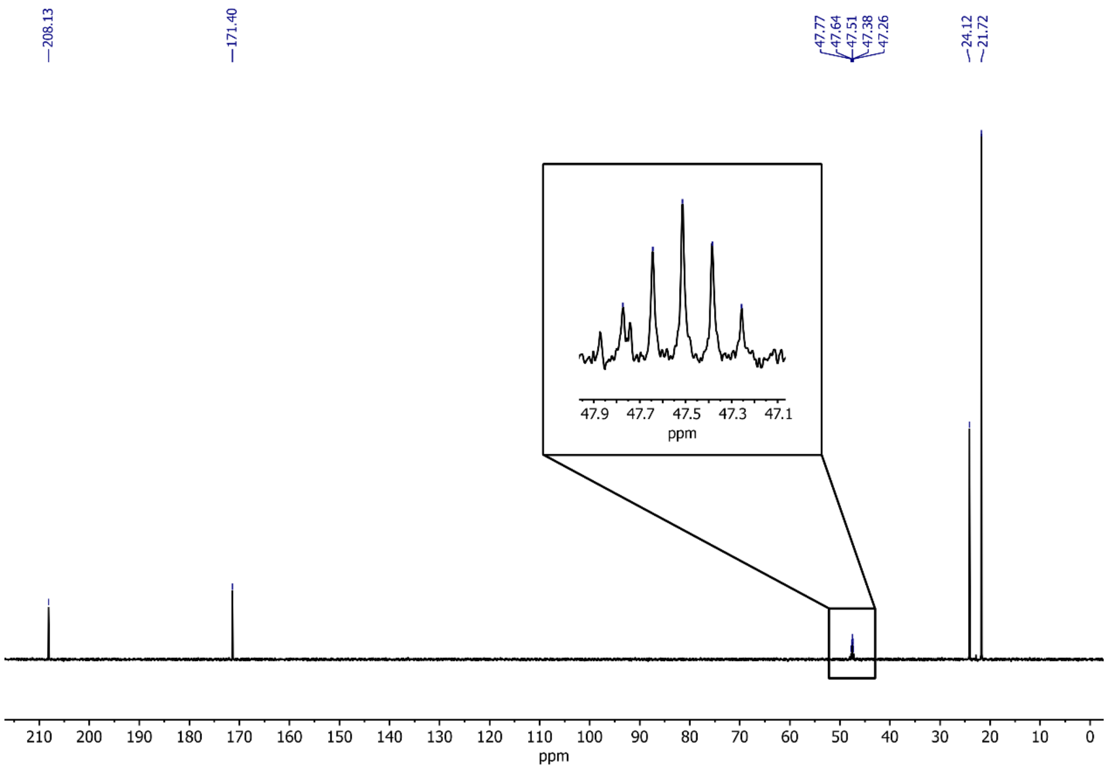

Figure S6: ^{13}C NMR (151 MHz, D_2O) of KIC-d_2.

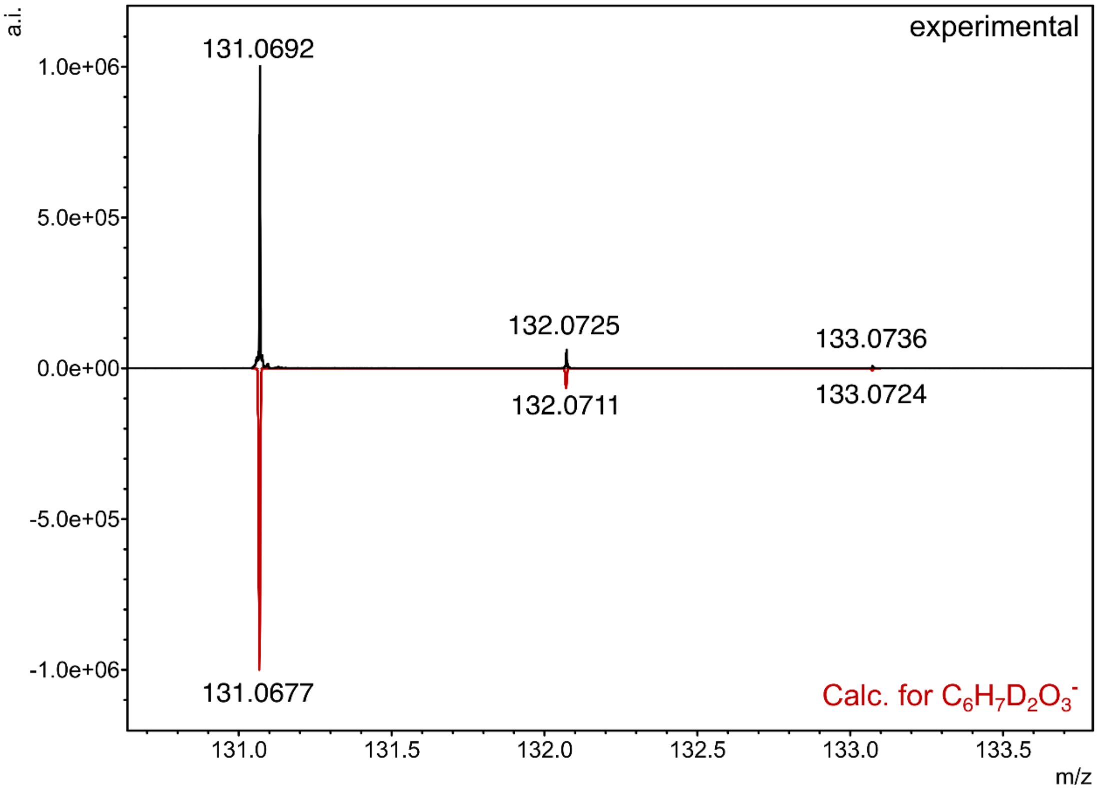

Figure S7: HRMS (ESI) of KIC-d_2.

Sample Preparation

Parahydrogen (pH_2) was produced and quantified using a previously described setup and method.[2,3] For optimization studies the pH_2 was produced using a liquid N_2 system, leading to 44 % parahydrogen. For all studies in the main paper (comparison of protonated and partially deuterated substrates, *in vitro*, and *in vivo* studies), pH_2 was produced using a Helium compressor and the enrichment was approximately 90 %.

The SABRE solutions were produced by mixing 30 mM [1-^{13}C]KIC, KIC-d_2 or [1-^{13}C]KIC-d_2 with 5 mM IrIMes(COD)Cl pre-catalyst (Cat), 23 mM deuterated dimethyl sulfoxide (DMSO-d_6, CAS: 2206-27-1), and 0.65 mM ethylenediaminetetraacetic acid (EDTA, CAS: 60-00-4) in degassed methanol-d_4 (Sigma Aldrich, 99.8 % D). For in vitro studies, the solution was diluted with methanol-d_4 to a final KIC concentration of 10 mM, whereas for in vivo experiments the concentration was adjusted to 50 mM [1-^{13}C]KIC-d_2, 9 mM Cat, 25 mM DMSO-d_6 and 0.3 mM EDTA. After 2-3 min sonication, the solutions were filtered using a 1.2 µm syringe filter.

For the purification, a phosphate buffered solutions (PBS) was prepared by dissolving commercially purchased phosphate buffer powder (pH 7.3-7.5, 0.017 g mL^{-1} in water, high purity, SKU P7994-1EA, Sigma Aldrich) in deionized D_2O (Sigma Aldrich, 99.9 % D).

Experimental Setup

The hyperpolarization experiments were carried out in a custom-built apparatus as previously described (Figure S8).[2] A resistive solenoid coil (length 360 mm, inner diameter 120 mm, two layers of 1.12 mm copper wire, 47 compensation windings at each end) generated the static magnetic field (B_0) along the z-axis. The radiofrequency field (B_1) required for SLIC polarization transfer was produced by a saddle-shaped coil (one winding around a 320 mm long, 100 mm inner diameter tube) and controlled via a custom Python program interfaced with a digital-to-analog converter (DAC, NI USB-6251, National Instruments, USA). The output signal was amplified using a 12 W audio amplifier (KEMO M032S) before transmission to the B_1 coil. To suppress external magnetic perturbations, the system was enclosed in a three-layer µ-metal shield (ZG-209, Magnetic Shield Corp.). The homogeneity of B_0 and B_1 fields within the sample volume was better than ±1 %, verified using a fluxgate magnetometer (Fluxmaster, Stefan-Mayer Instruments, Germany).

^{13}C Hyperpolarization was performed in 5 mm medium-wall NMR tubes (Wilmad Labglass, USA) equipped with a custom gas-bubbling assembly based on established designs.[4–6] A water bath maintained a constant sample temperature of 7 °C during the experiments.

NMR measurements for quantifying T_1, polarization level and *in vitro* studies were acquired on a 1 T benchtop spectrometer at 38 °C (Spinsolve Carbon 43, Magritek, Germany). Additional T_1 measurements were performed on a 1.4 T benchtop NMR (Spinsolve Carbon 60, Magritek, Germany) and thermal concentration quantification measurements on a 9.4 T NMR (AvanceNeo 400 MHz WB, Bruker, Germany). *In vivo* studies were conducted on a 7 T preclinical MRI scanner (BioSpec 70/20, PV6.0.1, Bruker, Germany) using a dual-tuned ^{1}H/^{13}C quadrature-quadrature volume coil (d = 35mm, V-XLS-HL-070-01349 V01, Rapid Biomedical, Germany).

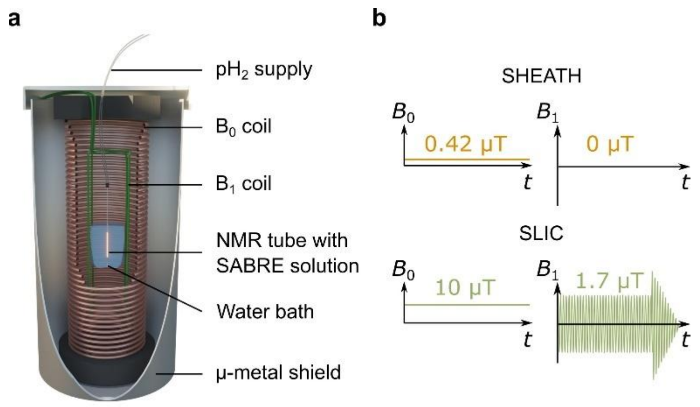

Figure S8: **a** SLIC-SABRE apparatus comprising μ-metal magnetic shielding, orthogonal B_0 and B_1 coils, an NMR tube immersed in a water bath, and pH_2 delivery via catheter. **b** Magnetic-field regimes used for SABRE-SHEATH and SLIC-SABRE polarization transfer.

Hyperpolarization and Purification Experiments

600 µL SABRE solution were filled into the NMR tube and bubbled for 3 minutes at 9.5 bar with pH_2 to activate the catalyst. For hyperpolarization the samples were placed into the SABRE polarizer with the respective field and temperature conditions to enable the spin order transfer from pH_2 to the ^{13}C of the substrate (optimizations in Figure S9-S11). For SABRE-SHEATH a constant B_0 field of 420nT was applied. SLIC-SABRE was performed at various B_0 fields between 5 µT and 200 µT with an oscillating B_1 field at the corresponding field-dependent ^{13}C Larmor frequency (e.g. ω=535.25 Hz at 50µT). The B_1 amplitude (≈1.7µT) was optimized for each B_0 field condition due to a slightly frequency dependent audio amplifier. After the SLIC pulse, an adiabatic 90° pulse aligned the magnetization back to the z-axis by gradually decreasing the B_1 amplitude from 4 µT to 0 µT within 2 seconds, while increasing the B_1 frequency by 50 Hz.

The purification procedure was performed using an adapted protocol based on previous publication (Figure S13).[2,7,8] 600 µL of pH-buffered D_2O was added to the SABRE solution, and methanol-d_4 was removed by evaporation at approximately 100 mT magnetic field, 10 mbar pressure, and 100 °C in 16 s. The buffer was applied in two steps, 300 µL at the beginning of evaporation, 300 µL after 11 s. The resulting mixture was filtered to remove precipitated iridium catalyst, yielding an aqueous D_2O solution of [1-^{13}C]KIC-d_2 (pH 7.0-7.5) suitable for in vivo administration. The residual methanol concentration was 212 ± 74 mM, measured by ^2H NMR.

Hyperpolarization Quantification

The ^{13}C polarization was calculated by using a thermally polarized external ^{13}C reference sample (neat [1-^{13}C]acetic acid, c_{13C} = 17.4 M, CAS: 1563-79-7, 99 atom % ^{13}C, Sigma Aldrich) using following equation:

$$P_{hyp} = \frac{I_{hyp}}{I_{ref}} \cdot \frac{c_{ref}}{c_{hyp}} \cdot \frac{N_{ref}}{N_{hyp}} \cdot \frac{f_{ref}}{f_{hyp}} \cdot P_{ref}$$

I denotes the integrated signal, *c* the concentration, *f* the ^{13}C enrichment, and *N* the number of averages (for both measurements N = 1) of the hyperpolarized "*hyp*" or reference sample "*ref*". P_{ref} is the thermal polarization of the reference sample at room temperature (298 K) and 1 T magnetic field of the NMR system.

Cell handling

MDA-MB-231, MCF-7 and PyB6 313 cells were cultivated using DMEM (Gibco 41966-052) supplemented with 1% L-Glutamine (Sigma-Aldrich G7513) + 1% Penicillin/Streptomycin (Gibco 11548876) and 10% fetal bovine serum (PAN Biotech P30-3306) at 37°C and 5%CO_2 and where kindly supplied by Prof. Reinheckel. At 80-90% confluency, cells were harvested using 0.05% Trypsin-EDTA

(Gibco 25300054), counted and aliquoted to $7.5 \cdot 10^6$ cells. After centrifugation, the pellet was resuspended in 200 µL of cultivation medium and transferred into a 5 mm NMR tube using a syringe with a silica catheter (ID 150 µm) attached. The NMR tube was held in a 37°C water bath until injection of the purified SABRE solution and subsequent measuring.

^{13}C NMR measurements

Hyperpolarization optimization

Various parameters of the SLIC-SABRE hyperpolarization were optimized and compared to SABRE-SHEATH: B_0 field including build-up and relaxation time characterization using 44 % pH_2 and non ^{13}C labeled substrates (Figure S9), B_1 amplitude and frequency (Figure S10), and Temperature (Figure S11). Temperature played a key role in controlling substrate exchange with the Iridium catalyst: the highest free-substrate polarization was achieved at 7 °C, whereas lower temperatures led to increased signal remaining on the catalyst-bound species. An exemplary Relaxation curve for 30 mM [1-^{13}C]KIC-d_2, hyperpolarized using SLIC-SABRE at 10 µT is shown in Figure S12.

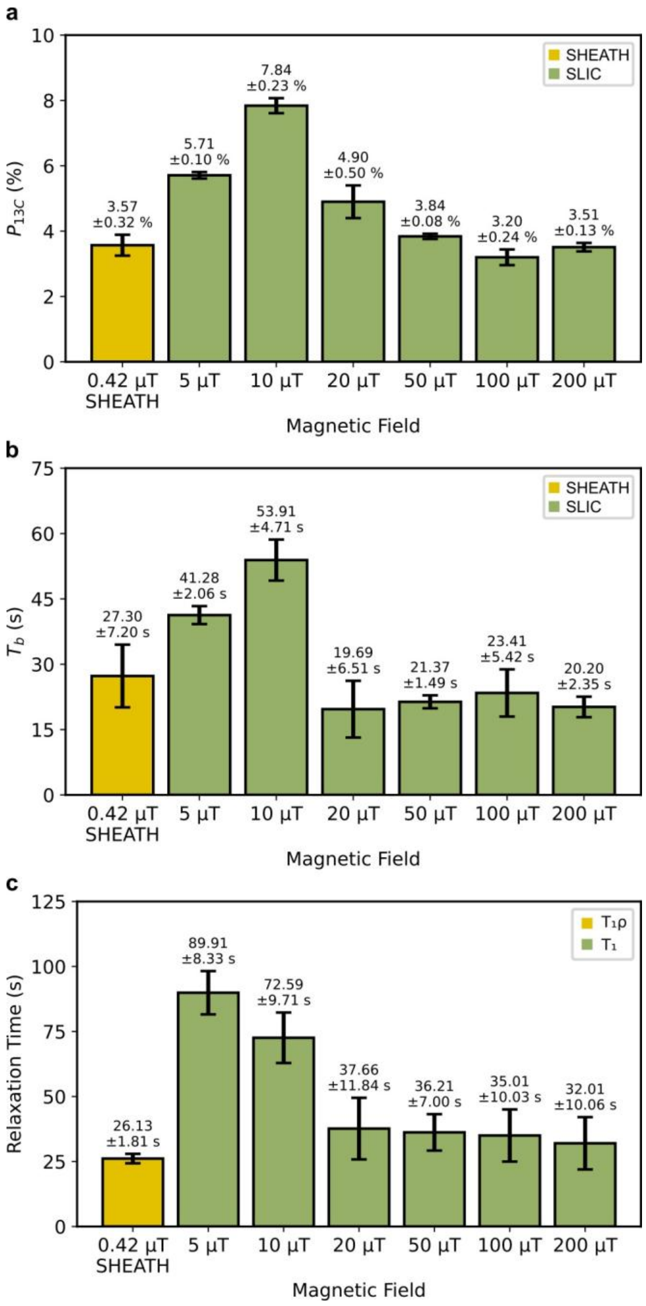

Figure S9: B_0-field optimization and characterization of SLIC-SABRE hyperpolarization compared with SABRE-SHEATH for KIC-d_2 using 44 % parahydrogen enrichment. **a** Maximum ^{13}C polarization levels. **b** ^{13}C polarization build-up times. **c** Field-dependent longitudinal (T_1), and spin-lock ($T_{1ρ}$) relaxation times.

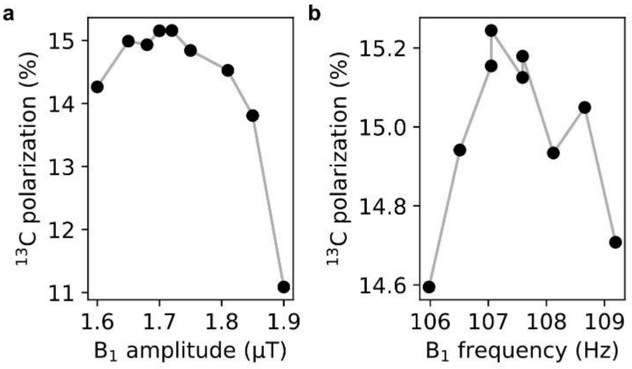

Figure S10: **a** Optimization of the B_1-field amplitude and **b** frequency of the SLIC pulse using [1-^{13}C]KIC-d_2 and a pulse duration of 60 s.

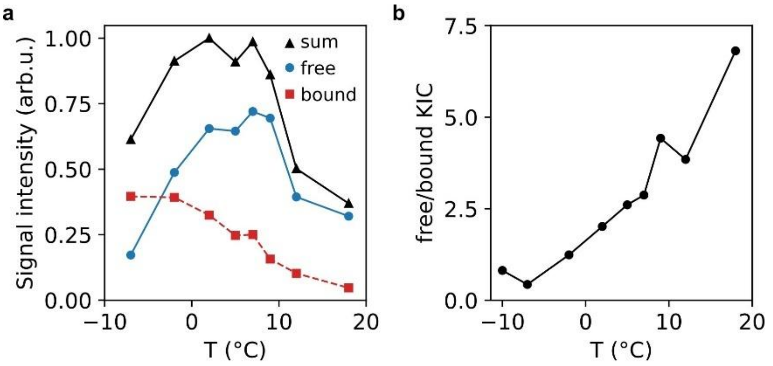

Figure S11: **a** Temperature dependence of the ^{13}C NMR signal intensity and the corresponding contributions from free and bound KIC-d_2 after 30 s of SLIC-SABRE hyperpolarization. **b** Ratio of the free-to-bound signal depending on the temperature.

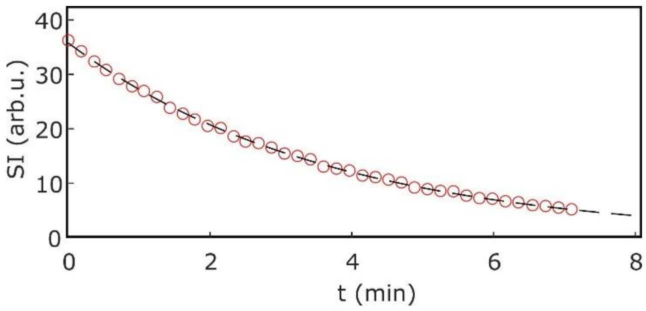

Figure S12: ^{13}C T_1 relaxation at 1 T for a 30 mM [1-^{13}C]KIC-d_2 solution.

Purification

The purification, adapted from [2], was further developed towards a two-step procedure, that leads to elevated substrate concentrations after purification (Figure S13). The substrate and methanol concentrations of the purified samples were determined by comparing known reference samples with the purified SABRE solution ^{13}C and ^{2}H NMR at 9.4 T (Figure S14). The purified samples were diluted with 200 µL cell medium.

The iridium concentration was quantified using an inductively coupled plasma–optical emission spectrometer (ICP-OES, PerkinElmer Avio 220 Max, Figure S15). Calibration was performed using a five-point external calibration curve. A single-element iridium standard solution was used to prepare the calibration standards (ROTI®Star ICP standard, 1000 mg·L^{-1} Ir in 10% HCl matrix; Carl Roth). A matrix-matched “blank” reference was prepared using the same matrix composition as the samples to minimize potential matrix effects.

For sample preparation, a 200 µL aliquot of each sample was acidified with 0.5 mL of 67% HNO_3 (ICP-MS grade). Subsequently, these samples were diluted to a final volume of 2 mL using deionized water (<5 MΩ·cm). Prior to analysis, all solutions were homogenized and allowed to equilibrate. The emission line of Ir at 212.681 nm was selected for quantification because of its sensitivity and freedom from spectral interferences. Each sample was measured in triplicate to ensure analytical precision.

A summary of the purified solutions characterization is provided in Table S1.

Exemplary relaxation time curves before and after purification are shown in Figure S16. A zoomed-in ^{13}C NMR spectrum of hyperpolarized KIC-d_2 solutions before and after purification is shown in Figure S17.

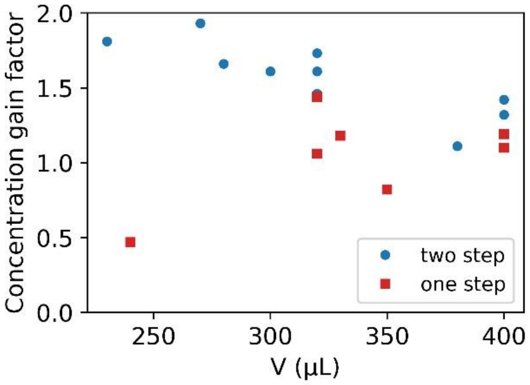

Figure S13: Comparison of the concentration gain via purification for various final sample volumes after purification using either a one-step or two-step PBS/D_2O injection protocol. In the one-step procedure, 600 µL of buffer were added at the start of purification; in the two-step procedure, 300 µL were added at the start and the remaining volume 5 s before completion.

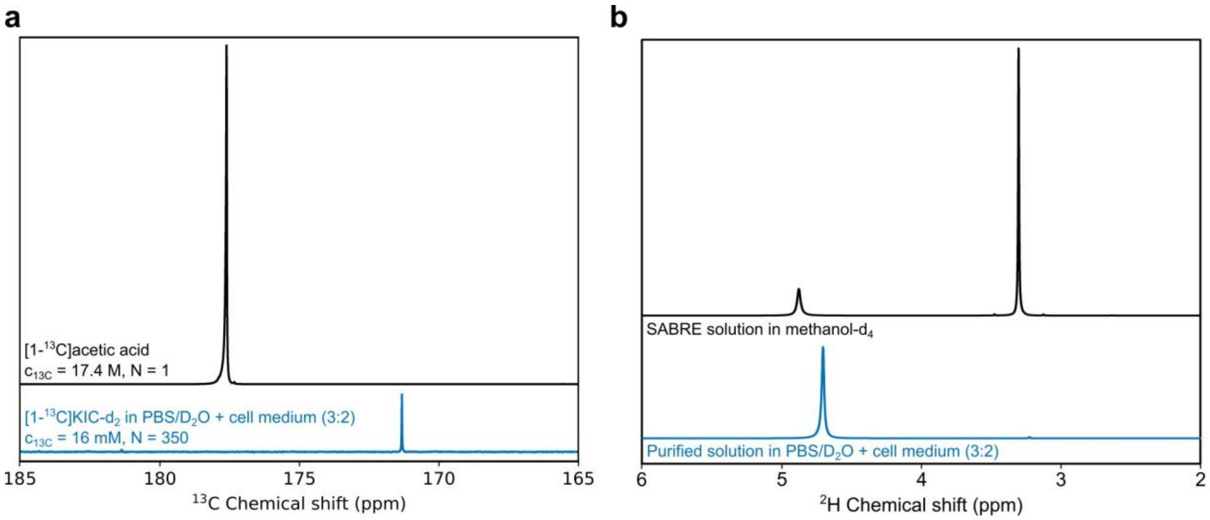

Figure S14: **a** Thermal ^{13}C NMR spectra of [1-^{13}C]KIC-d_2 after purification with PBS/D_2O, compared with neat [1-^{13}C]acetic acid to determine the substrate concentration after purification (dilution compensated: 16 mM). **b** Thermal ^2H NMR spectra of a [1-^{13}C]KIC-d_2 SABRE solution before purification in methanol-d_4 and after purification in PBS/D_2O allow the determination of residual methanol-d_4. The purified samples were diluted with 200 µL cell medium.

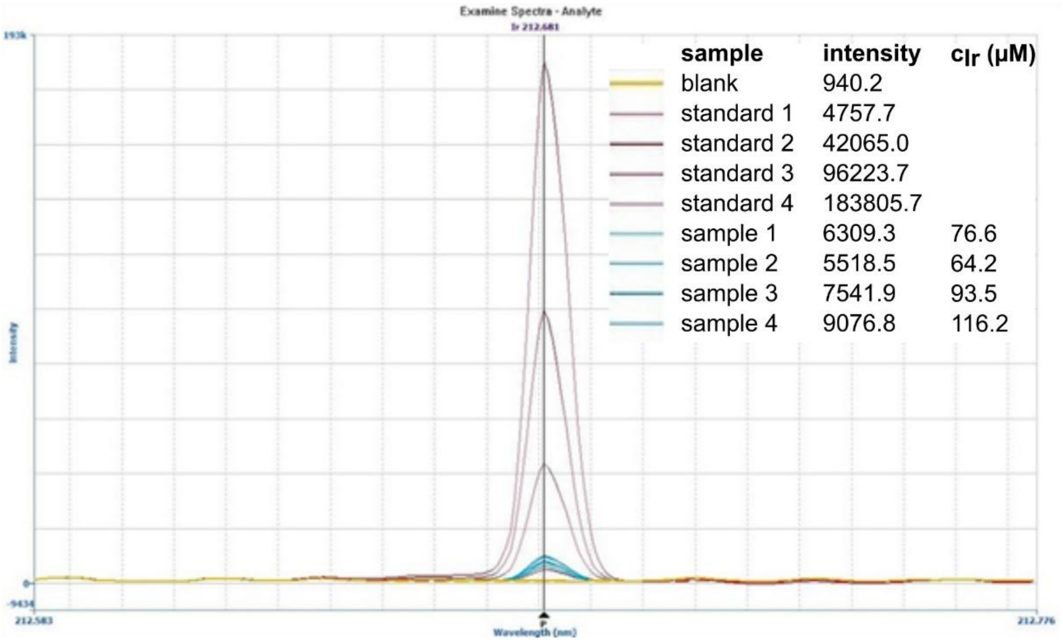

Figure S15: ICP-OES emission line at 212.681 nm for quantification of the iridium concentration in the purified solution.

	In vitro ($c_{KIC,initial}$ = 10 mM)	***In vivo*** ($c_{KIC,initial}$ = 50 mM)
$N_{samples}$	3	4
$c_{KIC,injection}$	16.3 ± 1.0 mM	52.7 ± 0.9 mM
$c_{methanol}$	212 ± 74 mM	370 ± 56 mM
c_{Ir}	Not quantified	88 ± 11 µM
**pH**	≈7.4	≈7.4
**V**	283 ± 27 µL	290 ± 17 µL
**P(^{13}C)**	9.5 ± 0.5 %	10.4 ± 0.6 %

Table S1: Characterization of the purified solutions used for *in vitro* and *in vivo* application.

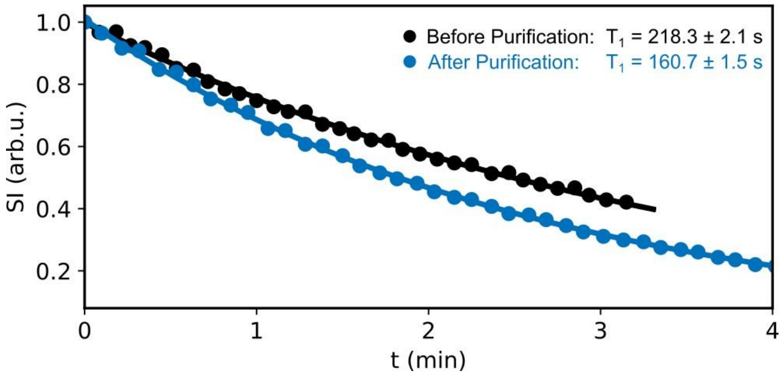

Figure S16: ^{13}C T_1 relaxation at 1 T for a 50 mM [1-^{13}C]KIC-d_2 solution before purification in methanol-d_4 and after purification in PBS in D_2O.

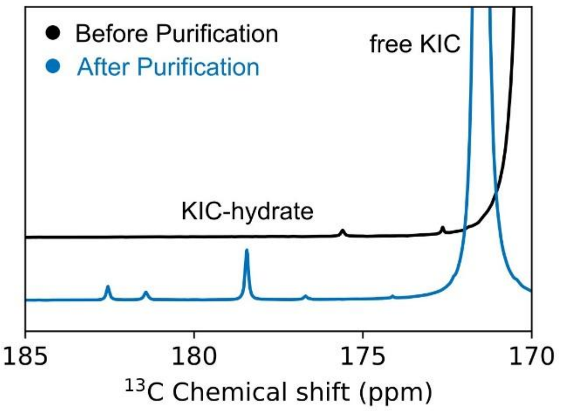

Figure S17: HP ^{13}C NMR spectra of a 50 mM [1-^{13}C]KIC-d_2 solution before and after purification show the appearance of KIC-hydrate at 178.3 ppm and additional two weak resonances at ≈ 182 ppm.

In Vitro Experiments

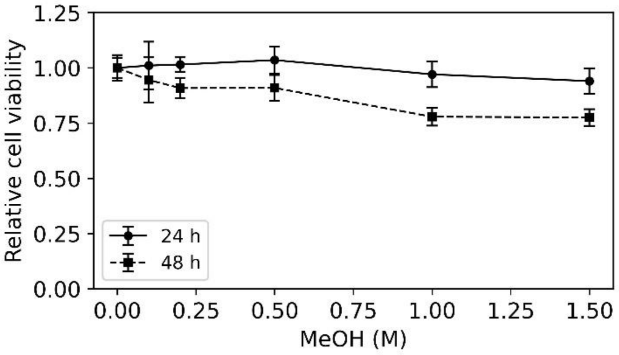

Figure S18: Cytotoxicity of methanol at varying concentrations, measured by MTT assay after 24 h and 48 h. Cell (PyB6-313 murine breast cancer cells) viability remains unaffected at low methanol concentrations and decreases only at concentrations above 500 mM. Data represents one biological replicate with three technical replicates per condition; values are normalized to untreated controls (0 M methanol). The data was previously published in [2].

Cell lines

All of the used cell lines are breast cancer cell lines of different origin. The PyB6 cells were isolated from a breast tumor of the MMTV-PyMT mouse model (C57BL/6 background) and immortalized *in vitro* by serial passaging.[9] The MDA-MB231 as well as MCF7 cell lines are both from human origin and represent different subtypes of breast cancer.[10] While MDA-MB231 cells represent triple-negative breast cancer, MCF7 cells present with a luminal phenotype.

Western Blot

The cell lines were subjected to protein isolation by RIPA buffer (1.5 M NaCl, 0.5 M EDTA (pH 7), 250 mM $Na_4P_2O_7$, 100 mM β-Glycerophosphate, 1% Triton-X-100, 0.001 g ml^{-1} SDS, 0.005 g ml^{-1} sodium deoxycholate, 1 mM sodium orthovanadate, 1x/10 ml PhosStop and 1x/10 ml cOmplete ULTRA in 50 mM Tris, pH 7.4) and mechanic forces on ice. Protein concentration was measured using the Pirece BCA Protein Assay Kit (Thermo Scientific) and 25 µg of protein/sample was loaded on gels for SDS-PAGE. Separated proteins were transferred on a nitrocellulose membrane in a wetblot and blocked with 3% BSA in 0.1% PBST for 1 h before incubating with either BCAT1- (88785, Cell signaling) or BCAT2-targeting (79764, Cell Signaling) antibody and an antibody targeting TUBA (66031, Proteintech) as loading control overnight at 4°C. BCAT1 and corresponding TUBA control were detected using HRP-

coupled antibodies with SuperSignal™ West Pico PLUS Chemiluminescent Substrate (Thermo Scientific) in the Fusion (Vilber). In contrast, BCAT2 and corresponding TUBA control were detected using fluorescently labelled antibodies in the Odyssey CLx (LICOR). Measured band intensities were normalized to the loading control TUBA.

Interestingly, the used cell lines show differences in their BCAT expression. On protein level, BCAT1 was only detected in MDA-MB231 cells (Figure S19a, indicated by an arrow), whereas no band was observed for MCF7 as well as for PyB6 cells. Interestingly, BCAT2 protein expression was highest in MCF7 cells, followed by MDA-MB231 cells and PyB6 cells showing no band for BCAT2 (Figure S19d, indicated by an arrow).

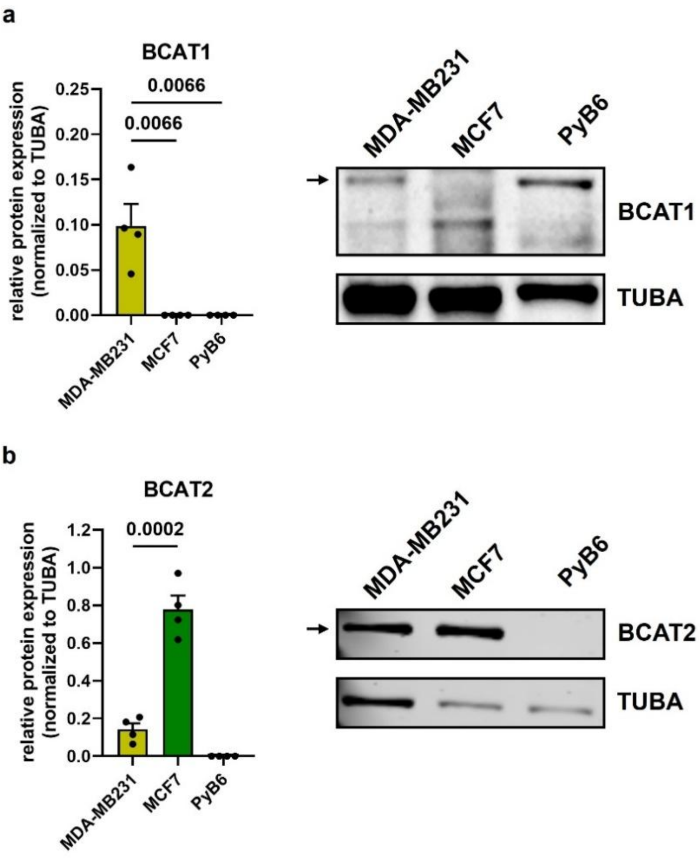

Figure S19: a BCAT1 protein expression in MDA-MB231, MCF7 and PyB6 cells (N=4, unpaired t-test). **b** BCAT2 protein expression in MDA-MB231, MCF7 and PyB6 cells (N=4, unpaired t-test). The arrows are indicating the BCAT1/2 bands.

In Vivo Experiments

The experimental procedures followed internationally accepted recommendations and guidelines for the handling of laboratory animals. Ethical approval for the animal experiments reported here was obtained from the relevant authority and experiments were conducted as described in the approval (Regierungspraesidium Freiburg, Talstr. 4-8, 79095 Freiburg; AZ: 35-9185.81/G-24/133). C57BL/6N mice (female, ≈25 g, ≈40 weeks old) with no genetic modifications were used for the study. Anesthesia was induced using isoflurane (2-4% in >99.5% O2, ≈1.0 L min^{-1} during spontaneous breathing) and the animal's vital signs, breathing and temperature, were continuously monitored (SA Instruments 1030, Stony Brook, NY 11790). To maintain a respiration rate of about 70 min^{-1}, the anesthesia depth was adjusted as needed. Respiration was monitored using a pressure-sensitive cushion. The animal's body temperature was monitored using a rectal thermometer and stabilized to 37 °C using a custom-made

water circulation system driven by a water pump. The mice were humanely euthanized via cervical dislocation under anesthesia at the end of the experiment. The bolus of 50 mM hyperpolarized KIC solution was administered within ≈5 s through the tail vein (total volume injected 10 µL g^{-1} mouse weight).

MRI Sequences and Data Processing

^{1}H T_2w RARE MRI was acquired for anatomical reference using TR = 6000 ms, TE = 6.3 ms, RARE factor = 12, FOV = 25 × 25 mm², matrix 256 × 256, slice = 1 mm, BW = 100 kHz.
^{13}C MRI (Figure 4a) was acquired using 2D FID chemical shift imaging (FIDCSI) using TR = 85 ms, α = 15°, FOV = 25 × 25 mm², matrix of 8 × 8, slice = 12 mm, center frequency of 175 ppm, spectral bandwidth of 33 ppm (2500 Hz, 200 points). The Image was acquired 15 s post-injection.
Dynamic ^{13}C MRS (Figure 4b, 4c) was acquired using TR = 5 s, α = 15°, center frequency of 175 ppm, spectral bandwidth of 79.6 ppm (6009 Hz, 200 points).
The metabolite maps were generated by integration (KIC: 170.5 ppm - 174 ppm, LAC: 175.5 ppm - 176.5 ppm) of the Fourier-transformed ^{13}C spectra (5 Hz line broadening) in each voxel. The maps displayed in main text Figure 4a were 4x interpolated using the MATLAB function 'imresize' with bicubic interpolation and normalized to a saturation value. A transparency cut-off was added (lower end in the color bars in the shown ^{13}C MRI images) to achieve optimal visualization of both anatomy and metabolism.
For spectrum processing, the free induction decay (FID) was apodized with 5 Hz exponential line broadening, zero-filled by a factor of 2, Fourier transformed with FFT-shift, corrected by zero- and first-order phase adjustment, and second-order polynomial baseline corrected using a custom Python script.

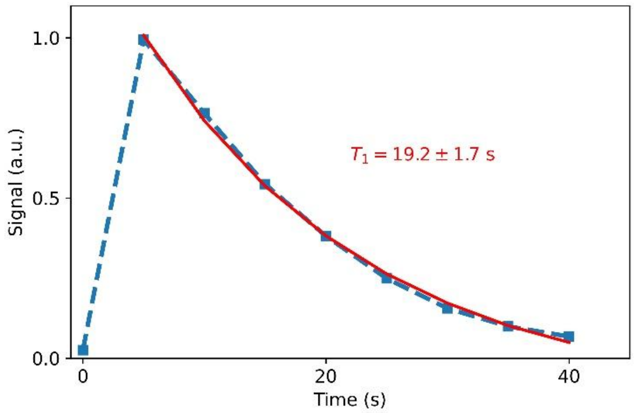

Figure S20: Curve fitting to extract the apparent *in vivo* ^{13}C T_1 relaxation of 50 mM [1-^{13}C]KIC-d_2 at 7 T.

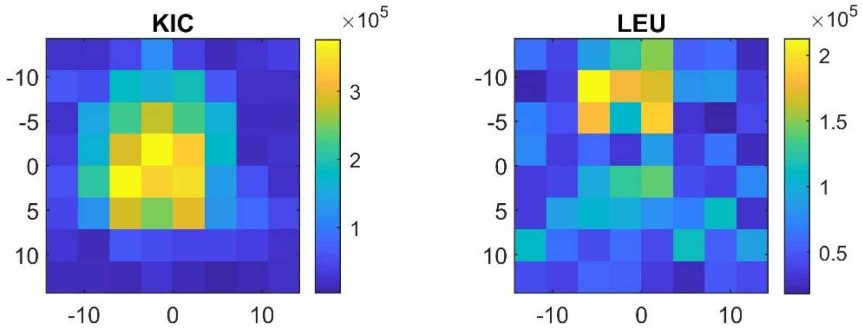

Figure S21: Non-interpolated ^{13}C KIC and LEU images based on integration in each voxel.

References

[1] R. Savka, H. Plenio, "Facile synthesis of [(NHC)MX(cod)] and [(NHC)MCl(CO)2] (M = Rh, Ir; X = Cl, I) complexes" *Dalton Trans.* **2014**, *44*, 891–893.

[2] H. de Maissin, P. R. Groß, O. Mohiuddin, M. Weigt, L. Nagel, M. Herzog, Z. Wang, R. Willing, W. Reichardt, M. Pichotka, L. Heß, T. Reinheckel, H. J. Jessen, R. Zeiser, M. Bock, D. von Elverfeldt, M. Zaitsev, S. Korchak, S. Glöggler, J.-B. Hövener, E. Y. Chekmenev, F. Schilling, S. Knecht, A. B. Schmidt, "In Vivo Metabolic Imaging of [1-13C]Pyruvate-d3 Hyperpolarized By Reversible Exchange With Parahydrogen**" *Angew. Chem. Int. Ed.* **2023**, *62*, e202306654.

[3] J.-B. Hövener, S. Bär, J. Leupold, K. Jenne, D. Leibfritz, J. Hennig, S. B. Duckett, D. von Elverfeldt, "A continuous-flow, high-throughput, high-pressure parahydrogen converter for hyperpolarization in a clinical setting" *NMR Biomed.* **2013**, *26*, 124–131.

[4] A. S. Kiryutin, G. Sauer, S. Hadjiali, A. V. Yurkovskaya, H. Breitzke, G. Buntkowsky, "A highly versatile automatized setup for quantitative measurements of PHIP enhancements" *J. Magn. Reson.* **2017**, *285*, 26–36.

[5] A. B. Schmidt, H. de Maissin, I. Adelabu, S. Nantogma, J. Ettedgui, P. TomHon, B. M. Goodson, T. Theis, E. Y. Chekmenev, "Catalyst-Free Aqueous Hyperpolarized [1-13C]Pyruvate Obtained by Re-Dissolution Signal Amplification by Reversible Exchange" *ACS Sens.* **2022**, *7*, 3430–3439.

[6] S. Nantogma, M. R. H. Chowdhury, M. S. H. Kabir, I. Adelabu, S. M. Joshi, A. Samoilenko, H. de Maissin, A. B. Schmidt, P. Nikolaou, Y. A. Chekmenev, O. G. Salnikov, N. V. Chukanov, I. V. Koptyug, B. M. Goodson, E. Y. Chekmenev, "MATRESHCA: Microtesla Apparatus for Transfer of Resonance Enhancement of Spin Hyperpolarization via Chemical Exchange and Addition" *Anal. Chem.* **2024**, *96*, 4171–4179.

[7] S. Petersen, L. Nagel, P. R. Groß, H. de Maissin, R. Willing, L. Heß, J. Mitschke, N. Klemm, J. Treiber, C. A. Müller, S. Knecht, I. Schwartz, M. Weigt, M. Bock, D. von Elverfeldt, M. Zaitsev, E. Y. Chekmenev, J.-B. Hövener, A. F. Martins, F. Schilling, T. Reinheckel, A. B. Schmidt, "*In vivo* molecular imaging of breast cancer metabolic heterogeneity using [1-^{13}C]pyruvate-d$_3$ hyperpolarized by reversible exchange with parahydrogen" *Theranostics* **2025**, *15*, 3714–3723.

[8] Y. Ding, S. Korchak, S. Mamone, A. P. Jagtap, G. Stevanato, S. Sternkopf, D. Moll, H. Schroeder, S. Becker, A. Fischer, E. Gerhardt, T. F. Outeiro, F. Opazo, C. Griesinger, S. Glöggler, "Rapidly Signal-enhanced Metabolites for Atomic Scale Monitoring of Living Cells with Magnetic Resonance" *Chemistry–Methods* **2022**, *2*, e202200023.

[9] S. Ketterer, J. Mitschke, A. Ketscher, M. Schlimpert, W. Reichardt, N. Baeuerle, M. E. Hess, P. Metzger, M. Boerries, C. Peters, B. Kammerer, T. Brummer, F. Steinberg, T. Reinheckel, "Cathepsin D deficiency in mammary epithelium transiently stalls breast cancer by interference with mTORC1 signaling" *Nat. Commun.* **2020**, *11*, 5133.

[10] C. Gest, U. Joimel, L. Huang, L.-L. Pritchard, A. Petit, C. Dulong, C. Buquet, C.-Q. Hu, P. Mirshahi, M. Laurent, F. Fauvel-Lafève, L. Cazin, J.-P. Vannier, H. Lu, J. Soria, H. Li, R. Varin, C. Soria, "Rac3 induces a molecular pathway triggering breast cancer cell aggressiveness: differences in MDA-MB-231 and MCF-7 breast cancer cell lines" *BMC Cancer* **2013**, *13*, 63.
